# Cortical Spreading Depression Promotes Persistent Mechanical Sensitization of Intracranial Meningeal Afferents: Implications for the Intracranial Mechanosensitivity of Migraine

**DOI:** 10.1523/ENEURO.0287-16.2016

**Published:** 2016-12-22

**Authors:** Jun Zhao, Dan Levy

**Affiliations:** 1 Department of Anesthesia, Critical Care and Pain Medicine, Beth Israel Deaconess Medical Center, Boston, 02215 MA; 2 Harvard Medical School, Boston, 02115 MA

**Keywords:** afferent, cranial meninges, headache, mechanosensitization, migraine, trigeminal

## Abstract

Migraine is one of the most common and disabling diseases in the world. A major feature of migraine headache is its aggravation by maneuvers that momentarily increase intracranial pressure. A key hypothesis implicates mechanical sensitization of trigeminal afferents that innervate the intracranial meninges in mediating this feature of migraine. However, whether such pain-related neural response actually develops under endogenous conditions that are linked specifically to migraine remains to be established. Single-unit recordings in the trigeminal ganglion of anesthetized male rats were combined with quantitative mechanical stimulation of the cranial dura mater to determine whether cortical spreading depression (CSD), an endogenous migraine-triggering event, affects the mechanosensitivity of meningeal afferents. CSD gave rise to an almost threefold increase in the magnitude of the responses to mechanical stimuli in 17 of 23 of the afferents tested. CSD-evoked meningeal afferent mechanosensitization occurred with a delay of 23.1 ± 2.2 min and lasted 64.1 ± 6.8 min in recording sessions that lasted for 90 min and for 177.5 ± 22.1 min in recording sessions that were extended for 240 min. Some of the sensitized afferents also developed a shorter-lasting increase in their ongoing discharge rate that was not correlated with the increase in their mechanosensitivity, suggesting that CSD-evoked meningeal afferent sensitization and increase in ongoing activity are independent phenomena. These novel findings support the notion that mechanical sensitization of meningeal afferents serves as a key nociceptive process that underlies the worsening of migraine headache during conditions that momentarily increase intracranial pressure.

## Significance Statement

Migraine headache is associated with symptoms suggestive of exaggerated intracranial mechanosensitivity. Enhanced mechanosensitivity of meningeal afferents could mediate this migraine feature, but whether such neural response occurs under endogenous conditions linked specifically to migraine remains a matter of speculation. Elicitation of cortical spreading depression (CSD), an endogenous migraine trigger, led to a pronounced and persistent increase in the mechanosensitivity of meningeal afferents that was not correlated with the additional shorter-lasting increases in the afferents’ ongoing activity. Mechanosensitization of meningeal afferents, induced by CSD and possibly other migraine triggers, could serve as a key nociceptive process that underlies the intracranial pain of migraine headache and its worsening during conditions that momentarily increase intracranial pressure, such as rapid head movements and coughing.

## Introduction

Migraine is the third most prevalent and seventh most disabling disease in the world, affecting about 15% of the adult population worldwide (Stovner et al., 2007; Steiner et al., 2013). Although the exact biological conditions underlying migraine remain unclear, the head pain of migraine is believed to be mediated by trigeminal primary afferent neurons that innervate the cranial meninges and their related large vessels (Messlinger, 2009; Noseda and Burstein, 2013). One of the key features of migraine pain points to the presence of increased intracranial mechanosensitivity, similar to the headaches that accompany certain intracranial pathologies, in particular aggravation of the pain by maneuvers that momentarily increase intracranial pressure such as coughing, straining, bending over, or rapid head movement (Blau and Dexter, 1981). One mechanism that was proposed to play an important role in mediating this key feature of the migrainous headache is enhanced mechanosensitivity (i.e., mechanical sensitization) of intracranial trigeminal meningeal afferents (Strassman et al., 1996; Strassman and Levy, 2006; Olesen et al., 2009). Previous studies documented the development of mechanical sensitization in meningeal afferents in response to direct stimulation of their receptive fields (RFs) using exogenous application of pain-producing inflammatory agents that are not specific to migraine (Strassman et al., 1996; Levy and Strassman, 2002, 2004). Thus, it remains unknown whether such meningeal afferent sensitization can also develop under endogenous conditions that are linked specifically to migraine.

Cortical spreading depression (CSD), an abnormal self-propagating slow wave of neuronal and glial depolarizations, has been proposed as the neural substrate of the abnormal visual symptoms (i.e., visual aura) that often precede the headache of migraine (Hadjikhani et al., 2001; Cao et al., 2002; Charles and Baca, 2013). CSD has been hypothesized to promote the activation of the meningeal sensory pathway and the ensuing headache of migraine (Moskowitz, 1984). The notion that CSD in rodents can provide an experimental platform to investigate neural mechanisms underlying migraine headache (Moskowitz et al., 1993) has led to important findings that implicate CSD as a trigger of meningeal afferent–evoked meningeal vasodilation and activation of the central headache pain pathway (Bolay et al., 2002; Zhang et al., 2010, 2011; Karatas et al., 2013; Zhao and Levy, 2015) and as such, a potential target for migraine pain treatment (Ayata et al., 2006).

Here*, in vivo* extracellular single unit recording of mechanosensitive meningeal afferents was combined with quantitative mechanical stimulation of the cranial dura mater to test for the first time the hypothesis that CSD is an important endogenous cortical process that can lead to the development of mechanical sensitization of trigeminal afferents that innervate the intracranial meninges. The results suggest that CSD can promote a pronounced and persistent increase in the mechanosensitivity of meningeal afferents. The data further suggest that the mechanisms underlying the development and maintenance of meningeal afferent mechanical sensitization and those responsible for the increase in the afferents’ ongoing activity after CSD are distinct. The development of mechanical sensitization of meningeal afferents after CSD further substantiates the role of these trigeminal sensory neurons in mediating migraine headache. Mechanical sensitization of meningeal afferents could serve as a key neural process that underlies the worsening of the headache during conditions that momentarily increase intracranial pressure, such as rapid head movements and coughing.

## Materials and Methods

### Animals

Male Sprague-Dawley rats (250–350 g) were used throughout the study. All animal experiments were conducted in accordance with the experimental protocol approved by the institutional Animal Care and Use Committee.

### Surgery and electrophysiological recordings

Animals were deeply anesthetized with urethane (1.2–1.5 g/kg, i.p.). Core temperature was kept at 37–38°C using a homoeothermic control system. Animals breathed spontaneously room air enriched with O_2_. Physiological parameters were collected throughout the experiments, and data were collected only from animals exhibiting physiological levels of oxygen saturation (>95%), heart rate (350–450 bpm), and end-tidal CO_2_ (3.5–4.5%). With a saline-cooled dental drill, one craniotomy was made to expose the left transverse sinus as well as the adjacent cranial dura extending ∼2 mm rostral to the sinus. Another small burr hole (0.5-mm diameter) was made to expose a small area of dura above the frontal cortex to allow the induction of CSD. The exposed dura was bathed with a modified synthetic interstitial fluid containing 135 mm NaCl, 5 mm KCl, 1 mm MgCl_2_, 5 mm CaCl_2_, 10 mm glucose, and 10 mm HEPES, pH 7.2. Single-unit activity of meningeal afferents (1 unit/rat) was recorded in the ipsilateral (left) trigeminal ganglion using a 50- to 100-kΩ platinum-coated tungsten microelectrode (FHC, Bowdoin, ME). To avoid the induction of uncontrolled CSDs in the ipsilateral cortex, the recording electrode was advanced into the left ganglion through a contralateral angled approach, which spares the ipsilateral cortex. Meningeal afferent neurons were identified by their constant latency response to single shock stimulation applied to the dura above the ipsilateral transverse sinus (0.5-ms pulse, 5 mA, 0.5 Hz). The response latency was used to calculate conduction velocity (CV), based on a conduction distance to the trigeminal ganglion of 12.5 mm (Strassman et al., 1996). Neurons were classified as either C units (CV ≤ 1.5 m/sec) or Aδ units (1.5 < CV ≤ 5 m/s). All meningeal afferents tested were mechanosensitive when probed with von Frey filaments (0.03–6.9 g, Stoelting Co., Chicago, IL), and had at least 1 RF located on the left transverse sinus or its vicinity (<1 mm). Neural activity was digitized, and a real-time waveform discriminator (Spike 2 software, CED, Cambridge, UK) was used to create and store a template for the action potential evoked by electrical stimulation, which was used later to acquire and analyze the ongoing activity of the neurons and the activity evoked by mechanical stimulation and CSD.

### Detection of mechanical sensitization

Mechanical responsiveness was quantitatively determined in each afferent by recording the responses to mechanical stimuli (100-ms rise time, 2-s width, 120-s interstimulus interval) delivered using a feedback-controlled mechanical stimulator (Series 300B, Aurora Scientific, Aurora, ON, Canada) and a custom-written script for Spike 2. Stimulus trials for testing changes in mechanosensitivity included one threshold stimulus (TH, which normally evoked a 1- to 3-Hz response) followed by a suprathreshold stimulus (STH, usually ×2 of the threshold; 8- to 10-Hz responses) and were delivered every 15 min throughout the experiment. These parameters were used to avoid potential desensitization to the mechanical stimuli. Ongoing afferent discharge rate was recorded continuously between the stimulation trials. Baseline ongoing activity and responses to mechanical stimulation were determined during at least four consecutive trials before the elicitation of CSD. Only units that exhibited consistent responses (variation of <0.5 Hz for TH responses and <1.5 Hz for STH responses) during baseline recordings were tested further.

### Induction and monitoring of CSD

In each experiment, a single CSD episode was induced in the frontal cortex by pinpricking the cortex with a fine glass micropipette (diameter 10 μm) at ∼2 mm depth for 2 s. CSD was induced in the frontal cortex to avoid potential damage to the meningeal tissue near the RF of the studied afferents, which could have led to their sensitization. The occurrence of a CSD episode was determined noninvasively by recording simultaneously changes in cerebral blood flow (CBF) using laser Doppler flowmetry, with the probe positioned within the craniotomy, just above (1 mm) the exposed dura, near (∼1 mm) the RF of the recorded unit. Induction of CSD was considered successful when the typical hemodynamic signature characterized by a large transient (∼1- to 2-min) cortical cerebral hyperemia, followed by persistent (>1-h) post-CSD oligemia (Fordsmann et al., 2013), was observed.

### Data analyses

Offline analyses for afferent responses were conducted using template matching in Spike 2. Average data are presented as the mean ± SEM. Average data in figures is presented as the mean ± 95% confidence interval (CI). A neuron was deemed sensitized only if the following criteria were fulfilled: TH and/or STH responses increased to a level greater than the upper endpoint of the 95% CI calculated for the baseline mean; sensitization began during the first 60 min post-CSD; and sensitization lasted for at least 30 min (i.e., two consecutive trials). CSD-evoked increases in afferents’ ongoing activity were considered if the firing rate increased above the upper end point of the 95% CI calculated for the baseline mean for >10 min. Group differences were analyzed using two-tailed, Fisher’s exact test. Statistical differences were analyzed using two-tailed unpaired *t*-test or Mann for normally distributed data and with the Mann–Whitney rank sum test when data failed the normality test (Kolmogorov–Smirnov test) or equal variance test. To examine correlations between neural activation and sensitization parameters, either Pearson or Spearman correlation coefficient tests were used based on data normality. Results were considered to be significant at *p* < 0.05. Superscript letters listed with *p*-values correspond to the statistical tests shown in Table 1.

**Table 1.** Statistical table

Line	Data structure	Type of test	Power of 25-75% Confidence interval
a	Normality test: passed (*p* > 0.99); Equal variance test: failed (*p* < 0.05)	Mann–Whitney rank-sum test	25–75% non–sensitized: 0.31–0.95; sensitized: 0.16–0.5
b	Normality test: passed (*p* > 0.99); Equal variance test: passed (*p* = 0.5)	Unpaired *t* test	*p* = 0.66
c	Normality test: passed (*p* = 0.18); Equal variance test: failed (*p* < 0.05)	Mann–Whitney rank-sum test	25–75% non–sensitized: 0.01–0.155; sensitized: 0.05–0.84
d	Normality test: passed (*p* > 0.99); Equal variance test: passed (*p* = 0.08)	Unpaired *t* test	*p* = 0.77
e	Normality test: passed (*p* > 0.99); Equal variance test: passed (*p* = 0.75)	Unpaired *t* test	*p* = 0.87
f	Normality test: passed (*p* = 0.09); Equal variance test: passed (*p* = 0.58)	Unpaired *t* test	*p* = 0.77
g	Normality test: passed (*p* = 0.66); Equal variance test: passed (*p* = 0.98)	Unpaired *t* test	*p* = 0.38
h	Normality test: passed (*p* > 0.99); Equal variance test: passed (*p* = 0.57)	Unpaired *t* test	*p* = 0.28
i	Normality test: passed (*p* = 0.8); Equal variance test: passed (*p* = 0.67)	Unpaired *t* test	*p* = 0.69
j	Normality test: passed (*p* = 0.84); Equal variance test: failed (*p* < 0.05)	Mann–Whitney rank-sum test	25–75% Aδ: 0.45–2.32; C: 0.67–4.13
k	Normality test: passed (*p* = 0.11); Equal variance test: failed (*p* < 0.05)	Mann–Whitney rank-sum test	25–75% TH: 0.51–2.64; STH: 1.33–1.77
l	Normality test: passed (*p* = 0.06); Equal variance test: failed (*p* < 0.05)	Mann–Whitney rank-sum test	25–75% Aδ: 1.29–1.39; C: 1.41–1.83
m	Normality test: passed (*p* = 0.1); Equal variance test: passed (*p* = 0.75)	Pearson’s correlation coefficient test	*p* = 0.99
n	Normality test: passed (*p* = 0.08); Equal variance test: passed (*p* = 0.46)	Pearson’s correlation coefficient test	*p* = 0.99
o	Normality test: failed (*p* < 0.05); Equal variance test: failed (*p* < 0.05)	Spearman’s correlation test	25–75% activation: 1.46–4.25; TH: see ^k^
p	Normality test: failed (*p* = 0.06); Equal variance test: failed (*p* < 0.05)	Spearman’s correlation test	25–75% activation: see ^o^; STH: 1.33–1.64
q	Normality test: failed (*p* < 0.05); Equal variance test: failed (*p* < 0.05)	Spearman’s correlation test	25–75% activation: 17–40; TH: 30–90
r	Normality test: failed (*p* < 0.05); Equal variance test: passed (*p* = 0.1)	Pearson’s correlation coefficient test	*p* = 0.99

## Results

### CSD evokes a pronounced and persistent mechanical sensitization of meningeal afferents

**Figure 1.**
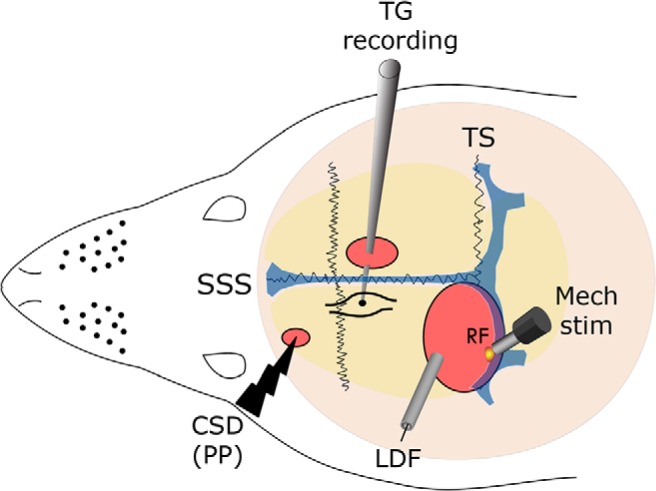
Experimental setup. Three skull openings (red ovals) were made. A small burr hole was made over the left frontal cortex to elicit CSD events using a pinprick (PP). Meningeal afferent activity was recorded in the left trigeminal ganglion (TG) using a tungsten microelectrode inserted through a craniotomy made over the contralateral hemisphere. An ipsilateral craniotomy was made to expose a part of the left transverse sinus (TS) and its vicinity to search for meningeal afferents with mechanical RF. Quantitative mechanical stimuli were delivered to the afferents’ RF using a feedback-controlled mechanical stimulator. Laser Doppler flowmetry (LDF) probe was placed over the cortex near the stimulated afferent’s RF to validate the induction of the CSD by testing related changes in cerebral blood flow. SSS, superior sagittal sinus.

The development of changes in mechanosensitivity of meningeal afferents in relation to the onset of CSD was studied by recording simultaneously the CSD-evoked CBF changes and single-unit activity in response to quantitative mechanical stimuli of the afferents’ meningeal RF (Fig. 1). The effect of CSD on the afferents’ responsiveness was investigated in 23 afferents (9 Aδ and 14 C-units). In control experiments, in which no CSD was induced, time-related changes in mechanosensitivity were examined in 12 meningeal afferents (5 Aδ and 7 C-units).

**Figure 2.**
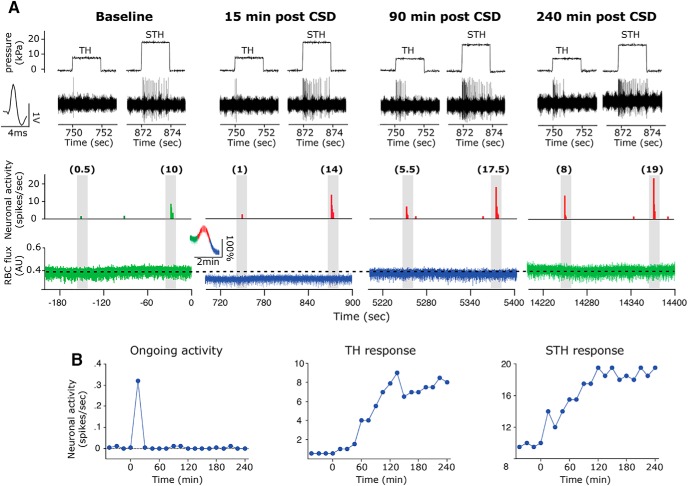
An example showing the development of mechanical sensitization after CSD in one C-unit meningeal afferent unit. ***A***, Top, trace examples of mechanically evoked afferent discharge to TH and STH stimuli during the last baseline stimuli trial, before the induction of CSD, and during the trials conducted at 15, 90, and 240 min after the induction of CSD. Below are matching peristimulus time histograms (PSTH, bean size 0.5 s) with mechanically evoked responses (spikes/s) in parentheses. The bottom trace illustrates the CBF at baseline and during the post-CSD mechanical stimulation trials. The insert denotes the acute changes in CBF during the arrival of the CSD near the RF of the recorded afferent. Note the CSD-evoked increase (red) and decrease (blue) in CBF. Also note the reduced CBF (blue traces) present at 15 and 90 min after the onset of CSD. ***B***, Time course data depicting the level of ongoing activity, TH and STH responses of the same unit during baseline sampling and every 15 min after the induction of CSD.

Single CSD events were successfully evoked in all cases where the frontal cortex was stimulated and were associated with a typical hemodynamic signature characterized by a brief (∼1-min) cortical cerebral hyperemia followed by a persistent (>1-h) post-CSD oligemia (Fordsmann et al., 2013; Gariepy et al., 2016; see also Fig. 2). After CSD, persistent mechanical sensitization, longer than 30 min of either TH and/or STH increased firing, was noted in 17 of 23 (∼74%) of the neurons (see an example in Fig. 2). We could not identify any significant differences between the response properties of afferents that became sensitized after CSD and of those that did not, including in baseline mechanosensitivity, number of distinct RFs, or baseline ongoing activity before the induction of CSD (Table 2, *p* > 0.05^a–c^ for all).

**Table 2.** Response properties of meningeal afferents that developed and did not develop mechanical sensitization following CSD

Sensitization	*n*	Baseline threshold (g)	Identified RFs	Baseline ongoing activity (Hz)
Sensitized	17	0.5 ± 0.2^a^	2.1 ± 0.3^b^	0.4 ± 0.1^c^
Nonsensitized	6	0.5 ± 0.2	2.0 ± 0.3	0.6 ± 0.3

Data show the mean ± SEM. (a–c) Two-tailed unpaired *t*-test revealed no significance differences between the groups.

In time control experiments, in which responses to mechanical stimuli were tested for at least 180 min, 5 of 12 (3 C-units, 2 Aδ) displayed a slight decline in mechanical responsiveness over time. In 6 of 12 units (3 Aδ, 3 C units), mechanical responsiveness remained stable over time, and in 1 of 12 units (C-unit) there was an increase in mechanical responsiveness (only at the TH level and for only 30 min). This frequency of sensitization in the control experiments was significantly lower than that observed after the induction of CSD (*p* < 0.001, Fisher’s exact test). Among the afferent population that developed mechanical sensitization response following CSD, there was no difference in the propensity to become sensitized between the Aδ (7/9) and the C units (10/14). These two neuronal populations also did not differ significantly with regard to the CSD sensitization rates at the TH or STH levels (Table 3), with most of the sensitized afferents (10/17) showing simultaneous sensitization at both the TH and STH levels.

**Table 3.** Rate of different types of mechanical sensitization responses in Aδ and C meningeal afferents following CSD

Group	TH only		STH only		TH + STH	
	Aδ	C	Aδ	C	Aδ	C
CSD	2/9 (11)	1/14 (7)	2/9 (22)	2/14 (14)	3/9 (30)	7/14 (50)
Control	0/5 (0)	1/7 (8)	0/5 (0)	0/7 (0)	0/5 (0)	0/7 (0)

Data show rate (% of responses). Sensitization at the TH and STH levels were determined according to the calculation described in the Methods. Two-tailed χ^2^ tests revealed no significant differences in the rate of the sensitization responses exhibited by the Aδ and C-unit populations.

**Figure 3.**
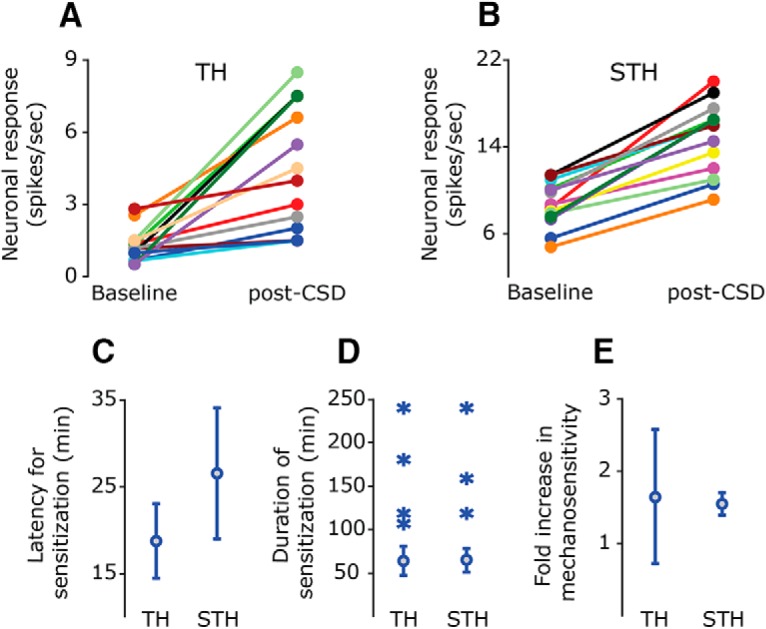
Summary of characteristics of the mechanical sensitization induced after the elicitation of CSD in the frontal cortex. TH (***A***) and STH (***B***) responses in neurons that exhibited mechanical sensitization. Data depict the mean responses at baseline, before CSD, and during the time of peak response after CSD (range 30–135 min). ***C***, Mean ± 95% CI of the latency to onset of persistent sensitization. ***D***, Duration of persistent sensitization. The means, indicated by circles (± 95% CI), reflect data from afferents in which CSD-evoked changes in mechanical responsiveness were studied for up to 90 min (*n* = 13). The durations of sensitization of units in which post-CSD responses were recorded for up to 240 min (*n* = 4) are indicated by asterisks. (E) Mean ± 95% CI of the magnitude increase in neuronal responses to TH and STH mechanical stimuli.

The onset latency of the CSD-induced mechanical sensitization ranged between 15 and 45 min, with most units (13/17) showing sensitization already at the first trial after CSD (i.e., at 15 min). As Fig. 3*C* depicts, the average onset latency of the TH sensitization response was 18.7 ± 1.9 min, and there was no significant difference between the sensitization latency of the Aδ units (18.7 ± 3.7 min) and the C-units (23.3 ± 5.1 min, *p* < 0.05^d^). The average latency of the STH sensitization response was 27.5 ± 3.6 min, which was not statistically different than that of the TH response (*p* > 0.05^e^). The STH sensitization onset latencies observed for the Aδ and C units were not statistically different (33.7 ± 3.8 vs. 22.5 ± 4.0 min, *p* > 0.05^f^).

In most afferent neurons, where the sensitizing effect of CSD was recorded for up to 90 min after the CSD, the overall duration of the TH sensitization responses ranged between 30 and 90 min and averaged 63.7 ± 7.6 min (Fig. 3*D*). There was no difference between the duration of the sensitization observed for the Aδ (72.0 ± 12.0 min) and C-unit (54.4 ± 9.4 min) populations (*p* > 0.05^g^). During the 90-min recording time period, the overall duration of the CSD-related sensitization at the STH level also ranged between 30 and 90 min and averaged 64.6 ± 6.4 min (*p* > 0.05 vs TH^h^) with no significant difference between the Aδ (57.0 ± 8.7 min) and C-unit (70.0 ± 7.9 min) populations (*p* > 0.05^i^). In four sensitized units, in which the post-CSD recording sessions were extended to 240 min (see an example in Fig. 2), mechanically evoked responses remained elevated for 105–240 min after CSD, with an average duration of 157.5 ± 33.3 min for the TH response and 202.5 ± 37.5 min for the STH response (see also Fig. 3*D*). In these units, the increased responsiveness was also observed during a time when the prolonged cerebral oligemia resolved and CBF returned to baseline levels.

As Fig. 3*E* depicts, during the sensitization state, the average increase in the TH response magnitude was 1.6 ± 0.4-fold (average peak magnitude 2.8 ± 0.9-fold). There was no significant difference (*p* > 0.05^j^) between the response magnitude of the Aδ units (average increase 1.5 ± 0.5-fold, peak increase 2.8 ± 1.2-fold) compared to the C-units (average increase 1.8 ± –0.6-fold, peak increase 2.9 ± 1.3-fold). The average increase in the magnitude of the STH responses was 1.5 ± 0.1-fold (peak increase 1.8 ± 0.1), which was not significantly different from that of the TH response (*p* > 0.05^k^). There was no difference between the average increase in mechanosensitivity noted for the Aδ and that noted for the C-units (1.3 ± 0.05-fold; peak response 1.5 ± 0.1-fold vs. 1.6 ± 0.1-fold; peak 2.0 ± 0.1-fold, *p* > 0.05^l^).

### Mechanical sensitization of meningeal afferents after CSD is not related to the development of increased ongoing activity

**Figure 4.**
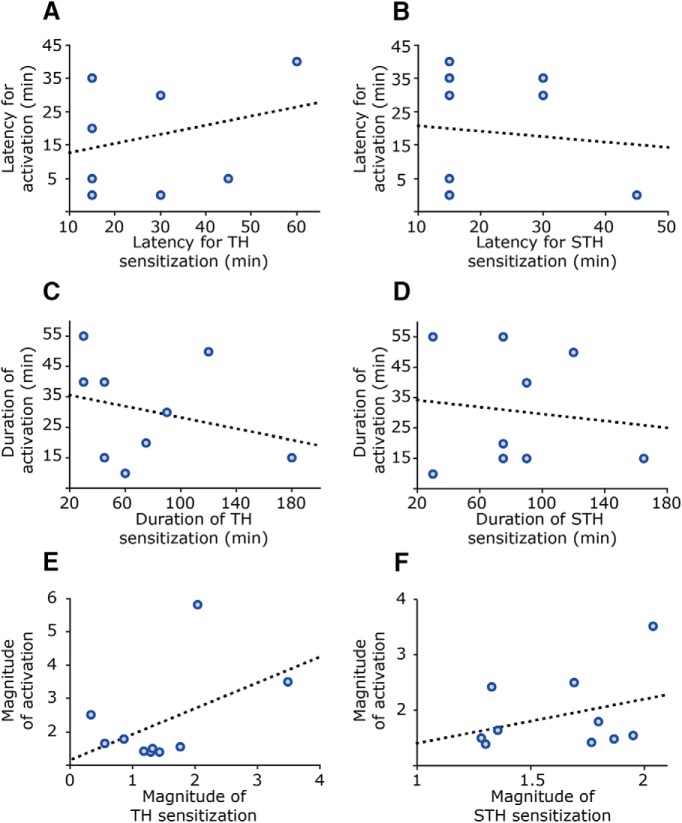
Mechanical sensitization of meningeal afferents induced after CSD is not correlated with the post-CSD increase in afferents’ ongoing activity. Pearson’s correlation indicated no linear relationship between the latency to onset of the sensitization and that of the increase in ongoing activity (***A***, ***B***). There was no significant correlation between the duration of the sensitization response and the duration of the increase in ongoing activity (***C***, ***D***). The magnitude of mechanical sensitization post-CSD was also not correlated with the magnitude of the increase in ongoing activity rate (***E***, ***F***).

To examine the possibility that the increase in ongoing activity and mechanical sensitization that develop following CSD are two unrelated processes, regression analyses were conducted to determine the correlation coefficients between the different parameters of the CSD-evoked activation and sensitization responses (Fig. 4*A–F*). Among the units that displayed both activation and sensitization, there was no correlation between the neural activation onset latency and the latencies for sensitization at either the TH level (regression coefficient *R* ^2^ = 0.06, *p* > 0.05^m^) or STH level (*R* ^2^ = 0.09, *p* > 0.05^n^). No correlation was found between the average magnitude of the activation response and that of the sensitization responses, at either the TH level (correlation coefficient *R* ^2^ = 0.23, *p* > 0.05^°^) or STH level (*R* ^2^ = 0.11, *p* > 0.05^p^). Finally, no correlations were found between the durations of the CSD-evoked neural activation and that of the sensitization responses at either the TH (regression coefficient, *R* ^2^ = 0.08, *p* > 0.05^q^) or STH (*R* ^2^ = 0.01, *p* > 0.05^r^) level. Further analyses of the sensitization and activation response durations revealed a longer duration for the mechanical sensitization response in comparison to the duration of the increase in ongoing activity; among all the units that exhibited an increase in ongoing activity, the duration of only 3 of 15 activated units exceeded 45 min. This relative rate was significantly lower than the rate observed for units that displayed a heightened mechanosensitivity at this time point, at the TH level (10/13; *p* < 0.01 by Fisher’s exact test) as well as at the STH level (10/14 units, *p* < 0.001 by Fisher’s exact test).

## Discussion

Mechanical sensitization of meningeal afferents has been hypothesized as a key nociceptive process that underlies the exacerbation of migraine headache during conditions that momentarily increase intracranial pressure, such as rapid head movements and coughing (Strassman et al., 1996; Strassman and Levy, 2006). The current data provide critical experimental evidence that supports this hypothesis by showing for the first time that CSD, a putative endogenous trigger of the migraine aura, is an important endogenous factor that can lead to the development of a persistent and pronounced increase in the mechanosensitivity of trigeminal afferents that innervate the cranial meninges.

Because migraine pain develops either during the aura phase or with a slight delay of ∼15 min (Hansen et al., 2012), the finding that mechanical sensitization could be observed in many meningeal afferents already at 15 min after CSD further supports the role of CSD as an important endogenous process that participates in the genesis of migraine headache. The finding that, after CSD, the sensitization of meningeal afferents could last for hours further substantiates the role of mechanosensitive meningeal afferents in mediating the onset of migraine headache as well as contributing to its persistence during the first hours of the attack.

The current data suggest that CSD promotes increased mechanosensitivity of meningeal afferents at the TH and STH levels. Increased mechanosensitivity around the afferents’ TH levels, which often also includes a reduction in their mechanical activation threshold (Levy and Strassman, 2002), may contribute to headache of migraine by allowing the afferents, in particular those that terminate on or very near meningeal blood vessels, to become activated in response to the small increase in the diameter of meningeal blood vessels seen during the attack (Amin et al., 2012, 2014) or the related stretching of the meninges. The development of meningeal vasodilatation during the headache stage may be due to an ongoing activation of meningeal afferents and the consequent release of vasodilating sensory neuropeptides, such as calcitonin gene–related peptide, through the process of neurogenic inflammation (Pietrobon and Moskowitz, 2012; Russo, 2015). The development of mechanical sensitization and the ensuing increased responsiveness to meningeal vessel dilatation and meningeal stretching may serve as a feed-forward mechanism that sustains the activity of the afferents and hence the headache. CSD-related sensitization of meningeal afferents at the STH level may particularly contribute to the exacerbation of the headache in response to conditions that promote transient increases in intracranial pressure, such as straining (Greenfield et al., 1984) and coughing (Williams, 1976).

In response to CSD, some afferents were sensitized only at either the TH or STH level, suggesting that these two processes occur independently. The cellular mechanisms that underlie mechanical sensitization at the TH and STH levels in general are not well understood but likely involve modulation of different ionic currents that control mechanotransduction and repetitive firing. Of note, it has been shown previously that activation of at least one biochemical cascade (the cAMP–PKA cascade) can result in differential effects on the TH and STH responses (Levy and Strassman, 2002). However, the finding that, after CSD, the majority of the sensitized afferents were affected at both the TH and STH levels suggests the involvement of multiple signaling cascades (Levy and Strassman, 2002).

In the present study, it was observed that numerous afferents developed mechanical sensitization in response to CSD together with an increase in their ongoing activity rate. The data analyses conducted suggest, however, that these phenomena are not related, pointing to the possibility of two distinct underlying mechanisms. It is also worth noting that the mechanical sensitization response after CSD lasted longer than the increase in ongoing activity, suggesting that CSD-evoked mechanical sensitization of meningeal afferents may play a more substantial role in the development of migraine headache. The cellular and molecular mechanisms that contribute specifically to the sensitization of meningeal afferents after CSD remain to be elucidated. The cortical depolarization that occurs during CSD gives rise to local release of numerous mediators with pro-nociceptive action, such as potassium, ATP, and arachidonic acid metabolites, into the interstitial space (Lauritzen et al., 1990; Schock et al., 2007; Enger et al., 2015). These and other algesic mediators such as nitric oxide (NO) may enter into the cerebrospinal fluid that circulates in the subarachnoid space (Shibata et al., 1991, 1992; Read et al., 1997), and if they reach a sufficient level, they could interact with meningeal afferents with RFs localized to the leptomeninges (Fricke et al., 1997, 2001), some of which may have collaterals that also terminate in the dura mater (O’Connor and van der Kooy, 1986; Kosaras et al., 2009). Interstitial mediators cleared via arachnoid granulations of the dural sinuses (Johnston et al., 2004) could act on meningeal afferents with RFs that terminate at these dural vascular locations. CSD-related parenchymal mediators that are cleared by the paravenous glymphatic pathway (Iliff et al., 2012) and subsequently through the dural lymphatic network (Aspelund et al., 2015; Louveau et al., 2015) could influence dural afferents with RFs that terminate at the wall of dural lymphatic vessels (Andres et al., 1987). Dural afferents may also become sensitized in response to a secondary event, such as dural neurogenic inflammation, as hypothesized earlier (Moskowitz, 1993). Among the CSD-related mediators, the highly diffusible NO is of particular interest given its ability to promote mechanical sensitization of meningeal afferents without an increase in their ongoing activity (Zhang et al., 2013). The release of cyclooxygenase metabolites, which mediate the persistent post-CSD cerebral oligemic responses (Shibata et al., 1992; Gariepy et al., 2016), may also contribute to the post-CSD afferent sensitization response (Levy et al., 2008). Importantly, however—because in some animals, mechanical sensitization was still present during the resolution of the oligemic response—it is unlikely that this cortical vascular response, in and of itself, is responsible for the persistence of the CSD-related mechanical sensitization.

In summary, the current study provides important in vivo data that further substantiate the role of trigeminal meningeal afferents in mediating migraine headache by showing that CSD, a putative migraine trigger, can lead to a pronounced and persistent sensitization of meningeal afferents. The development of mechanical sensitization of meningeal afferents, caused by CSD and perhaps other endogenous migraine triggering events, could serve as a key nociceptive process that mediates the exacerbation of the headache during conditions that momentarily increase intracranial pressure.

## Synthesis

Reviewing Editor: Karen Davis, Krembil Research Institute and University of Toronto Decisions are customarily a result of the Reviewing Editor and the peer reviewers coming together and discussing their recommendations until a consensus is reached. When revisions are invited, a fact-based synthesis statement explaining their decision and outlining what is needed to prepare a revision will be listed below. The following reviewer(s) agreed to reveal their identity: Gregory Dussor, Karl Messlinger.

This manuscript provides further characterization of the response properties of meningeal afferents following cortical spreading depression, an event thought to mediate migraine aura but one that is controversial regarding a role in migraine pain. Previously, it has been shown that CSD causes an increase in ongoing firing of meningeal afferents, with variable amounts of delay, but the current work describes the sensitization of these afferents to both threshold and suprathreshold mechanical stimulation. In anesthetized rats recordings were made from neurons in the trigeminal ganglion while cortical spreading depression (CSD) was released by mechanical injury of the cortical surface. The experiments show sensitization to mechanical stimulation after an approximately 20 min delay after CSD that lasts for between 60 and 180 min. Interestingly, while some afferents developed ongoing activity after CSD, there was no correlation observed between this activity and sensitization to mechanical stimulation in either latency, duration, or magnitude at either threshold or suprathreshold stimulus values suggesting separate processes contribute to each response. Thus, as previously shown (Zhang et al. 2010) CSD caused increased ongoing activity in most of the neurons and increased also responses to mechanical stimulation of the cranial dura mater. The demonstration of mechanical sensitization following CSD is new. It is speculated that similar mechanisms may underlie the hypersensitivity to mechanical stimuli and the throbbing nature of migraine pain. Together, the data provide further evidence of the sensitization of meningeal afferents by CSD and support the conclusion that CSD can contribute to the pain phase of migraine.

Sensitization to mechanical Stimulation of meninges following CSD is a new finding, but other phenomena (activation by chemical Stimulation and spontaneous activity) have been published. However, the manuscript shows that in addition to producing relatively short-lasting increases in ongoing afferent activity, cortical spreading depression is capable of producing longer-lasting sensitization of afferents to mechanical stimulation. The longer timeframe may be more relevant for the long-lasting headaches typical of migraine.

There are many issues to be addressed, but all are relatively minor:

1. Prior studies by Zhang et al 2010 used 3 different CSD induction methods, electrical stimulation, cortical pinprick, and KCl and there was variable duration of ongoing activity after CSD depending on the type of stimulus used (pinprick produced the shortest duration). The current work used pinprick for all experiments. Can the authors comment on why they chose pinprick, and whether they would expect any differential results had they chosen electrical stimulation or KCl, particularly in the correlation between ongoing and mechanical sensitization?

2. Additionally, prior work evoked CSD from the occipital cortex, while the current study stimulated the frontal cortex. Would location of induction lead to any differences in these data?

3. Figure 3 shows data out to 120 min but it seems that there were only 4 units that were studied for longer than 90 min, and those 4 went to 240 min. It is not clear why the figures go out to 120 min if most units stopped at 90 min but 4 went to 240 min. It would seem that the 90 and 240 min timepoints would be valuable to include, since 90 min is the latest timepoint that has all units included and 240 is the latest timepoint measured.

4. Figure 3 A and B are taken at the time point of peak response after CSD, according to the legend. Can the authors indicate what time point this is?

5. It seems as though there should be error bars on the data in Figure 2B.

6. Figure 2 should have a panel B but has a panel C. Also, the x-axis in B should be min not sec.

7. Methods, page 4, line 115: Please define "FHC"; line 116: typo "advances"; line 119: What means "units".

8. Please add stimulation parameters used for electrical stimulation of receptive fields for calculation of conduction velocities.

9. Page 6, lines 152-155: It seems that the sentence, as it stands, converts the events, namely persisting oligemia followed by short hyperemia.

10. Page 7, line 162: should probably be plural.

11. Results, page 8, line 187: typo "mechanosesnitivity

12. line 197: case should probably be imperfect

13. line 204: "A" in the bracket puzzling

14. line 207: How can the rate of activity in one unit be compared with that of the general sample using Fisher's test?

15. Discussion, page 11, lines 318-321: The throbbing nature of migraine headache has rather endogenous reasons than depends on mechanical stimulation through pulsating blood vessels, because the frequency of both phenomena does not match, as has been reported by Andrew Ahn (Headache 2010; 50: 1507-1510).

16. Discussion, page 13, lines 346-348: "The cortical depolarization that occurs during CSD gives rise to the release of numerous mediators with pronociceptive action such as potassium, nitric oxide (NO) ATP and arachidonic acid metabolites into the interstitial space". Please note that these substances may accumulate in the superficial cortex but not dura mater during CSD. It is very unlikely that they directly reach the primary meningeal afferents because of the subarachnoid space and a rather dense inner layer of the dura.

17. Figure 4EF: "Magnitide" on the abscissa seems to be a typo.
